# *HSD3B1* Expression Is Upregulated by Interleukin 4 in HT-29 Colon Cancer Cells via Multiple Signaling Pathways

**DOI:** 10.3390/ijms232113572

**Published:** 2022-11-05

**Authors:** Hsin-Mei Chen, Pei-Yu Hung, Chih-Hung Chen, Yu-Jhen Yu, Ming-Shan Syu, Meng-Chun Hu

**Affiliations:** Graduate Institute of Physiology, National Taiwan University College of Medicine, Taipei 10051, Taiwan

**Keywords:** HSD3B1, IL4, steroidogenesis, colon cancer

## Abstract

3β-Hydroxysteroid dehydrogenase/isomerase is essential for the synthesis of active steroid hormones. Interleukin 4 (IL4) induces the expression of *HSD3B1* in various human cancer cell lines. Here, we demonstrated that administration of IL4 to an HT-29 colon cancer cell line induced high expression of *HSD3B1* at the mRNA and protein levels. In the HT-29 cells, IL4 stimulated the activity of signal transducer and activator of transcription 6 (STAT6) and promoted its binding to the STAT6-binding site in the *HSD3B1* promoter. The STAT6 inhibitor significantly suppressed *HSD3B1* induction by IL4 in a dose-dependent manner. Moreover, inhibition of the PI3-kinase/AKT pathway strongly suppressed the IL4-induced *HSD3B1* expression. Glycogen synthase kinase 3 (GSK3), a downstream target of AKT, had a stimulatory effect on the IL4-induced *HSD3B1* expression. However, IL4 stimulated the phosphorylation of AKT, which inhibited the GSK3 activity at the early stage. Hence, GSK3 potentiated the *HSD3B1* levels at the late stage of the IL4 stimulation. Additionally, inhibitors of mitogen-activated protein kinases (MAPKs), ERK1/2 and p38, but not of JNK, partly reduced the *HSD3B1* expression following the IL4 stimulation. We further demonstrated that IL4 potently promoted steroid synthesis. Our results indicate that IL4 induces *HSD3B1* expression via multiple signaling pathways in HT-29 cells and may play a role in the regulation of steroid synthesis.

## 1. Introduction

Steroid hormones are predominantly produced by the endocrine glands, adrenal cortex, gonads, and placenta during pregnancy. They are synthesized from cholesterol via a series of enzymatic steps mediated by two enzyme families, the cytochrome P450 and hydroxysteroid dehydrogenase families [1]. Steroidogenic enzymes are also expressed in other organs, such as the thymus, brain, skin, intestines, and lungs, demonstrating a potential for local steroid synthesis [2,3,4]. The 3β-hydroxysteroid dehydrogenase/isomerase (3β-HSD) enzyme plays a pivotal role in the biosynthesis of all steroid hormone classes [5]. Two isoforms of 3β-HSD have been characterized in humans, type I and type II, which are products of hydroxy-delta-5-steroid dehydrogenase and of 3 beta- and steroid delta-isomerase 1 (*HSD3B1*) and 2 (*HSD3B2*), respectively. The adrenal cortex, ovaries, and testes express high mRNA levels of *HSD3B2,* which is responsible for the biosynthesis of glucocorticoids (GCs), mineralocorticoids, and sex steroids [6]. *HSD3B2* mutations are associated with a rare form of congenital adrenal hyperplasia [1]. *HSD3B1* is predominantly expressed in the placenta and is involved in the production of progesterone during pregnancy. Moreover, *HSD3B1* is expressed in several peripheral tissues, such as the skin and normal mammary glands [6]. Interleukin 4 (IL4) and IL13 induce 3β-HSD activity in normal human cells derived from mammary and prostate glands [7,8]. IL4 stimulates 3β-HSD activity in various cancer cells, including breast, cervical, and colon cancer cells [8]. However, IL4-induced *HSD3B1* expression is not observed in human choriocarcinoma cell lines.

IL4 is a Th2-associated cytokine produced by activated T cells, basophils, and mast cells. IL4 regulates the functions of T and B cells and immune processes. It promotes the differentiation of helper T cells into the Th2 type, induces the production of IgE by B lymphocytes, and plays a critical role in inflammation and tissue adhesion [9]. Elevated expression of IL4 and IL4 receptors has been demonstrated in various tumor cells, including lung, breast, pancreas, thyroid, and colon tumor cells [10,11,12,13]. IL4 protects cancer cells from cell death and chemotherapeutic drug-induced apoptosis [14,15]. The binding of IL4 to its receptor induces several intracellular pathways, including the signal transducer and activator of transcription 6 (STAT6) and insulin receptor substrate (IRS)-1/2 pathways [9,16]. STAT6 is phosphorylated by Janus kinases and forms dimers that translocate to the nucleus to activate the transcription of target genes. IL4 also stimulates the phosphorylation of IRS-1/2, which leads to the activation of the phosphoinositide-3-kinase (PI3K) and Ras/mitogen-activated protein kinase (MAPK) pathways [9,16]. A major downstream target of PI3K is serine/threonine kinase AKT, which is translocated to the inner membrane and phosphorylated by phosphoinositide-dependent kinase-1 [17]. Activated AKT phosphorylates many downstream substrates involved in the regulation of cell growth, survival, metabolism, and angiogenesis [18].

GCs are involved in various physiological processes, including stress response, metabolic activity, and immune regulation. Circulating GCs are predominantly secreted by the adrenal cortex and controlled by adrenocorticotropin released from the anterior pituitary [19]. Steroidogenic enzymes required for GC synthesis were found to be expressed in murine intestinal epithelial cells in response to immune stimulation and inflammation [20,21]. In a dextran sodium sulfate-induced mouse colitis model, intestinal GC synthesis and the expression levels of cytochrome P450 family 11 subfamily A member 1 (*Cyp11a1*) and subfamily B member 1 (*Cyp11b1*) were rapidly upregulated [22]. Locally synthesized GCs play a critical role in the regulation of intestinal immune homeostasis and may be linked to the pathogenesis of inflammatory bowel disease [20,22,23,24]. A decrease in the expression of steroidogenic genes is observed in the colonic epithelium of patients with ulcerative colitis [25]. Expression of steroidogenic genes and GC production also occur in colon cancer cell lines and human colon cancer specimens [26]. These tumor-derived GCs can inhibit T cell activation, which may suppress the immune response to facilitate tumor growth and metastasis.

IL4 is a critical regulator of intestinal immunity that exerts potent anti-inflammatory effects against intestinal inflammation [27,28,29]. IL4 stimulates 3β-HSD activity in colon cancer cell line HT-29 [8]; however, the regulation of IL4-mediated *HSD3B1* expression is not yet understood. In this study, we aimed to investigate the mechanism underlying IL4-regulated *HSD3B1* expression in HT-29 cells.

## 2. Results

### 2.1. IL4 Induces HSD3B1 Expression in HT-29 Cells

**Figure 1:**
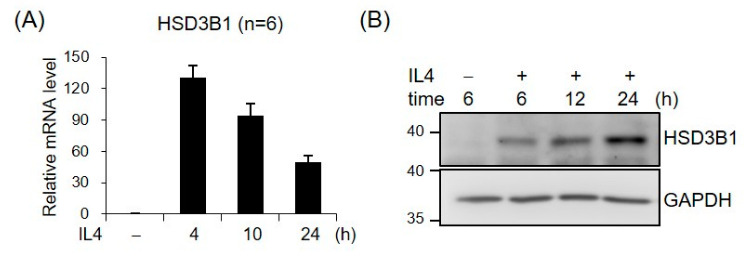
IL4 induces *HSD3B1* expression in HT-29 cells. The HT-29 cells were treated with IL4 (20 ng/mL) for the indicated period. (**A**) RNA was extracted, and *HSD3B1* mRNAs were quantified by real-time reverse transcription quantitative polymerase chain reaction (RT-qPCR). The values are represented as the means ± SEM of six independent experiments. (**B**) The cells were lysed and subject to immunoblotting for HSD3B1. GAPDH was used as the loading control.

To characterize the role of IL4 in *HSD3B1* expression, we examined the effects of IL4 on the mRNA and protein levels of *HSD3B1* over a 24 h time course in HT-29 cells. As shown in Figure 1, IL4 induced a robust increase in the expression of the *HSD3B1* mRNA and protein, as determined by real-time RT-qPCR and Western blotting analysis, respectively, while the kinetic patterns were notably distinct in HT-29 cells. The mRNA levels of *HSD3B1* dramatically increased to ~130-fold of the control within 4 h and then gradually decreased to ~50-fold of the control by 24 h. The HSD3B1 protein was expressed at relatively low levels in the HT-29 cells but was markedly induced after IL4 treatment for 6 h and continually increased to 24 h.

### 2.2. STAT6 Is Involved in IL4-Regulated HSD3B1 Expression in HT-29 Cells

**Figure 2:**
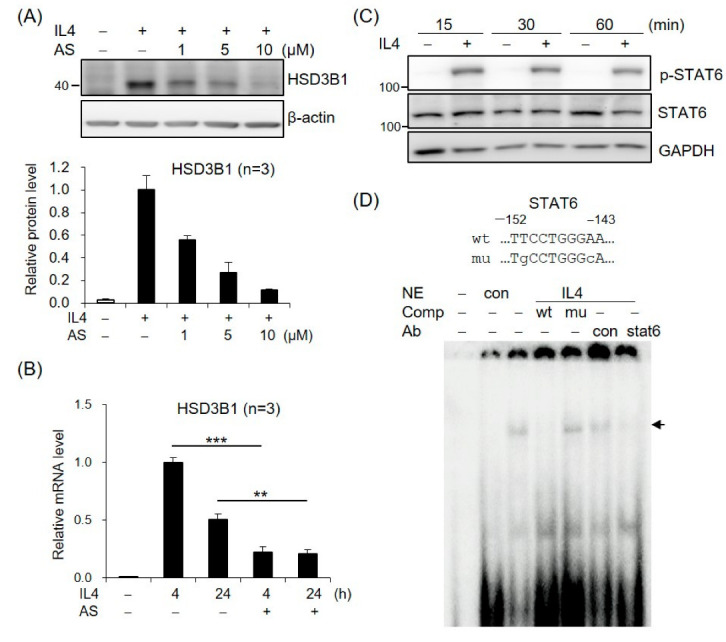
STAT6 is involved in IL4-induced *HSD3B1* expression in HT-29 cells. (**A**) The HT-29 cells were pretreated with STAT6 inhibitor AS1517499 (1, 5, or 10 μM) for 30 min and exposed to IL4 (20 ng/mL) for 24 h. The HSD3B1 protein levels were analyzed by immunoblotting and quantified by normalizing the β-actin level. The values are represented as the means ± SEM of three independent experiments. (**B**) The cells were pretreated with STAT6 inhibitor AS1517499 (10 μM) for 30 min and exposed to IL4 (20 ng/mL) for the indicated period. The *HSD3B1* mRNA levels were quantified by RT-qPCR. The values are represented as the means ± SEM of three independent experiments; ** *p* < 0.01, *** *p* < 0.001. (**C**) The cells were treated without or with IL4 (20 ng/mL) for the indicated period. The immunoblotting analysis showed both the phosphorylated and total STAT6 levels. (**D**) EMSA was performed with the nuclear extract (NE) from the HT-29 cells treated without or with IL4 (20 ng/mL) for 1 h. The probe contained a putative STAT6-binding site at −152/−143 in the *HSD3B1* promoter. Unlabeled competitors with or without mutations in the STAT6-binding site were used in 100-fold molar excess. Anti-STAT6 or control IgG was added in the binding reaction as indicated. The band of the STAT6–DNA complex is indicated by the arrow.

To assess the role of STAT6 in IL4-increased *HSD3B1* expression, we tested the effects of AS1517499, a selective STAT6 inhibitor. As shown in Figure 2A, treatment of the HT-29 cells with AS1517499 resulted in a significant inhibition of the IL4-induced HSD3B1 protein expression in a dose-dependent manner. Furthermore, the AS1517499 treatment decreased the IL4-induced *HSD3B1* mRNA levels to ~20% of the control value at 4 h (Figure 2B). The effect of IL4 on STAT6 activation was confirmed by immunoblotting with a phospho-specific antibody. Phosphorylation of STAT6 at Tyr641 was observed after 15 min of exposure to IL4 in the HT-29 cells (Figure 2C). EMSA with nuclear extracts from the IL4-treated HT-29 cells revealed that DNA–protein complexes were formed on oligonucleotides containing the STAT6-binding motif of the *HSD3B1* promoter (Figure 2D). The complexes were abolished by a 100-fold molar excess of the unlabeled probe but not by the mutated probe. Furthermore, the formation of IL4-induced complexes was disrupted by the addition of anti-STAT6 but not by control IgG (Figure 2D). These data demonstrate that STAT6 activation plays a critical role in the induction of *HSD3B1* expression by IL4 in HT-29 cells.

### 2.3. PI3K/AKT Signaling Is Involved in IL4-Regulated HSD3B1 Expression in HT-29 Cells

**Figure 3:**
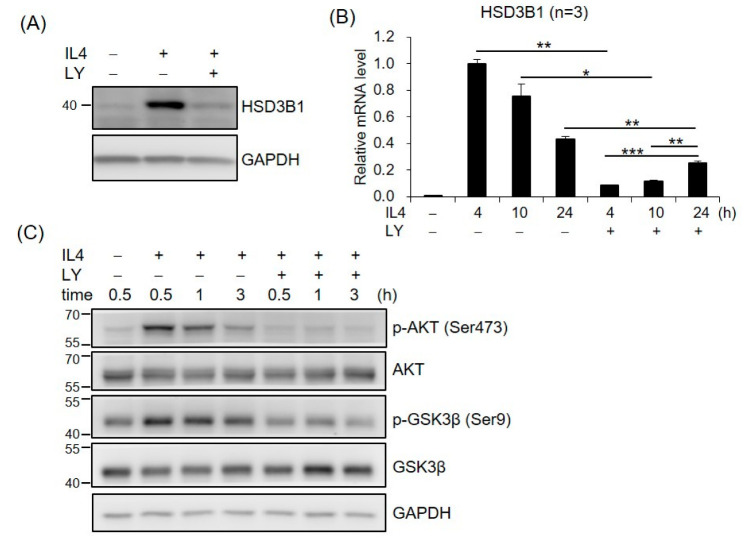
PI3K signaling is involved in IL4-induced *HSD3B1* expression in HT-29 cells. The HT-29 cells were pretreated with PI3K inhibitor LY294002 (LY, 20 μM) for 30 min and exposed to IL4 (20 ng/mL) for 24 h (**A**) or different time periods (**B**,**C**). (**A**,**C**) The cells were lysed and subject to immunoblotting using the indicated antibodies. GAPDH was used as the loading control. (**B**) RNA was extracted, and the *HSD3B1* mRNA levels were quantified by RT-qPCR. The values are represented as the means ± SEM of three independent experiments; * *p* < 0.05, ** *p* < 0.01, *** *p* < 0.001.

To investigate whether PI3K/AKT was required for IL4-induced *HSD3B1* expression, the effect of PI3K inhibitor LY294002 (LY) was examined in the HT-29 cells. As shown in Figure 3A, LY294002 markedly repressed the IL4-induced HSD3B1 protein expression in the HT-29 cells. Treatment of the HT-29 cells with LY294002 also resulted in a 91% inhibition of the IL4-stimulated *HSD3B1* mRNA levels at 4 h; however, this suppressive effect was attenuated at 24 h (42%) after IL4 stimulation (Figure 3B). We further examined the effects of IL4 on the activation of AKT, a major component of PI3K signal transduction, using Western blotting analysis with a phospho-specific antibody. In the HT-29 cells, phosphorylation of AKT at Ser473 was apparent 30 min after IL4 treatment and returned to nearly basal levels by 3 h (Figure 3C). PI3K inhibitor LY294002 blocked IL4 induction of AKT phosphorylation (Figure 3C). These results suggest an important role for PI3K–AKT signaling in the regulation of *HSD3B1* expression by IL4 in HT-29 cells. 

**Figure 4:**
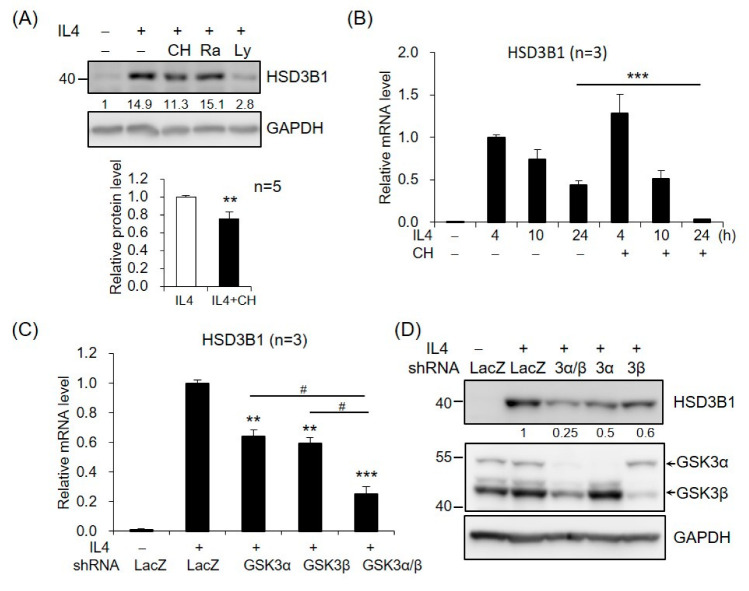
GSK3 is involved in IL4-induced *HSD3B1* expression in HT-29 cells. (**A**) The HT-29 cells were pretreated with GSK3 inhibitor CHIR99021 (5 μM), mTOR inhibitor rapamycin (25 μM), or PI3K inhibitor LY294002 (20 μM) for 30 min and exposed to IL4 (20 ng/mL) for 24 h. The HSD3B1 protein levels were analyzed by immunoblotting and quantified by normalizing the GAPDH level. The values are represented as the means ± SEM of five independent experiments; ** *p* < 0.01. (**B**) The cells were pretreated with CHIR99021 (5 μM) for 30 min and exposed to IL4 (20 ng/mL) for the indicated period. The *HSD3B1* mRNA levels were quantified by RT-qPCR. The values are represented as the means ± SEM of three independent experiments; *** *p* < 0.001. (**C**,**D**) The cells were transduced with shRNAs specific for GSK3α, GSK3β, or LacZ (negative control) for 48 h and exposed to IL4 for 24 h. The *HSD3B1* mRNA levels were quantified by RT-qPCR (**C**). The values are represented as the means ± SEM of three independent experiments; ** *p* < 0.01, *** *p* < 0.001 compared with shLacZ + IL4, ^#^ *p* < 0.05. (**D**) The protein levels of HSD3B1 and GSK3 were determined by immunoblotting. The numbers below the blot are the relative densitometric values normalized to those of GAPDH.

We further investigated the potential role of AKT downstream targets in *HSD3B1* expression in response to IL4 in HT-29 cells. GSK3 and mechanistic target of rapamycin kinase (mTOR) are two critical downstream regulators of the AKT kinase [18]. When the cells were treated with GSK3 inhibitor CHIR99021, the IL4-induced HSD3B1 protein expression was significantly reduced at 24 h (Figure 4A). In contrast, rapamycin treatment of mTORC1 had no effect on the HSD3B1 protein levels (Figure 4A). Further, CHIR99021 inhibited up to ~90% of the IL4-enhanced *HSD3B1* mRNA levels at 24 h, but it had no significant effect on the *HSD3B1* mRNA expression at the early timepoints (4 and 10 h) of IL4 stimulation (Figure 4B). To further verify the contribution of GSK3 in the regulation of IL4-induced *HSD3B1* expression, we performed shRNA knockdown experiments. GSK3 consists of two isoforms, GSK3α and GSK3β. The effectiveness of GSK3α and GSK3β knockdown was verified using RT-qPCR (Supplementary Figure S1). In the HT-29 cells, knockdown of GSK3α or GSK3β caused a marked reduction of the *HSD3B1* mRNA (Figure 4C) and protein (Figure 4D) levels by 40–50% after 24 h treatment with IL4. Simultaneous knockdown of GSK3α and GSK3β resulted in a 70–75% reduction in the IL4-induced *HSD3B1* expression. These data implicated that GSK3 activity positively regulates *HSD3B1* expression in IL4-stimulated HT-29 cells. GSK3 is ubiquitously expressed and constitutively active in cells. GSK3 activity is primarily inhibited by the phosphorylation of Ser21 in GSK3α and of Ser9 in GSK3β and mediated by several kinases, such as AKT [18,30]. As shown in Figure 3C, the levels of GSK3β phosphorylation at Ser9 were significantly increased after 0.5 h treatment with IL4 and then gradually declined, which correlated with the levels of AKT phosphorylation. Additionally, PI3K inhibitor LY294002 suppressed the IL4-induced phosphorylation of AKT and GSK3β. Therefore, GSK3 was inactivated upon the activation of AKT at the early stage of IL4 stimulation. This would explain the lack of inhibitory effects of the GSK3 inhibitor on the *HSD3B1* expression by IL4 at the early timepoints (Figure 4B). Subsequently, a reduced level of AKT phosphorylation led to a rise in GSK3 activity that enabled GSK3 to positively regulate the *HSD3B1* expression during the late period of IL4 stimulation.

### 2.4. MAPKs Are Involved in IL4-Regulated HSD3B1 Expression in HT-29 Cells

**Figure 5:**
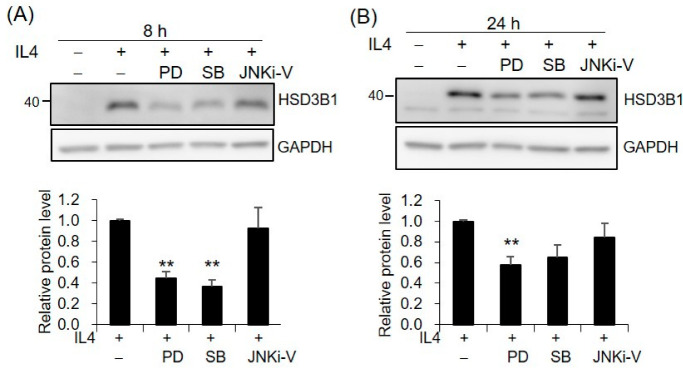
MAPKs are involved in IL4-induced *HSD3B1* expression in HT-29 cells. The HT-29 cells were pretreated with MEK1/2 inhibitor PD98059 (10 μM), p38 inhibitor SB202190 (2 μM), or JNK inhibitor JNKi-V (5 μM) for 30 min and exposed to IL4 (20 ng/mL) for 8 h (**A**) or 24 h (**B**). The HSD3B1 protein levels were analyzed by immunoblotting and quantified by normalizing the GAPDH levels. The values are represented as the means ± SEM of four independent experiments; ** *p* < 0.01.

Since MAPKs have been implicated in IL4 activity, we further investigated whether MAPKs are involved in the mechanism of IL4-induced *HSD3B1* upregulation. The contributions of the three major MAPKs, extracellular signal-regulated kinases 1/2 (ERK1/2), p38, and c-Jun N-terminal kinase (JNK), were determined using specific inhibitors PD98059, SB202190, and JNKi-V, respectively. PD98059 is an inhibitor of MAP kinase 1/2 (MEK1/2) that is the upstream kinase of ERK1/2. Pretreatment of the HT-29 cells with PD98059 resulted in ~56% and ~63% decreases in the IL4-enhanced HSD3B1 protein expression at 8 h and 24 h, respectively (Figure 5). Additionally, p38 inhibitor SB202190 showed 42% and 35% inhibition at 8 h and 24 h, respectively (Figure 5). However, no effect on the HSD3B1 protein induced by IL4 was observed with JNK inhibitor JNKi-V (Figure 5). To determine whether IL4 led to the activation of ERK1/2 and p38, the HT-29 cells were stimulated with IL4 and phosphorylated and total ERK1/2 and p38 were examined by immunoblotting analysis. We found that the phosphorylation of ERK1/2 and p38 was slightly affected by the IL4 treatment (Supplementary Figure S2A,C). Figure S2B showed that the phosphorylation of ERK1/2 was indeed suppressed by PD98059.

### 2.5. IL4 Induces 3β-HSD Activity and Bioactive GC Production in HT-29 Cells

**Figure 6:**
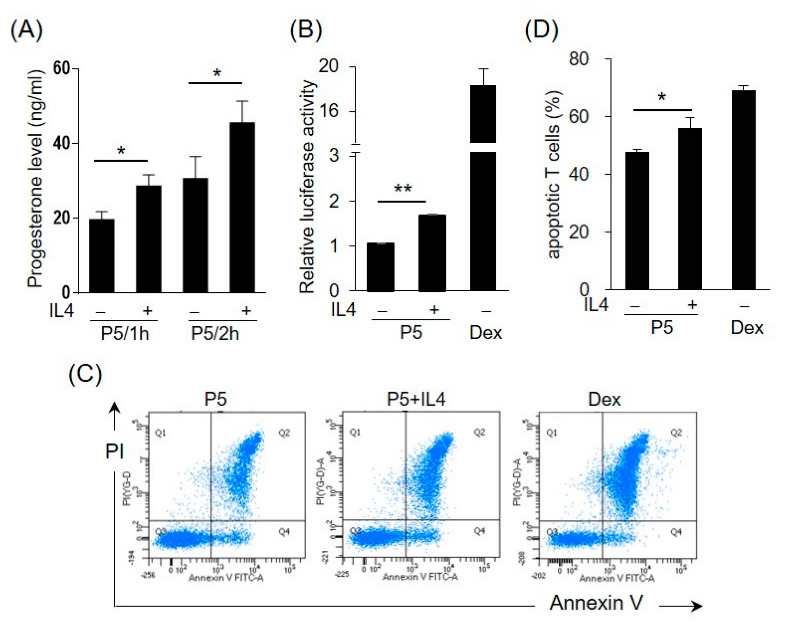
IL4 induces 3β-HSD activity and bioactive glucocorticoid (GC) production in HT-29 cells. (**A**) The HT-29 cells were treated with IL4 (20 ng/mL) or a vehicle for 12 h and the medium was replaced with a fresh medium containing pregnenolone (P5). After 1 or 2 h, the progesterone levels in the culture medium were measured. The values are represented as the means ± SEM of three independent experiments; * *p* < 0.05. (**B**–**D**) Conditioned media (CM) were prepared from the HT-29 cells treated with IL4 (20 ng/mL) or a vehicle with P5 for 24 h. Dexamethasone (10 nM in (**B**), 1 nM in (**D**)) was used as the positive control. (**B**) The pGRE-tk-Luc reporter construct was co-transfected into HEK293T cells with the expression vector for the GC receptor (GR). After 24 h, the cells were cultured in the HT-29-derived CM. The cells were harvested after 24 h and assayed for luciferase activity. The values are represented as the means ± SEM of three independent experiments; ** *p* < 0.01. (**C**) Murine splenocytes were isolated and cultured in the HT-29-derived CM. The murine splenic T cells were detected using an APC-conjugated anti-mouse CD 3e antibody. Apoptotic T cells were detected using annexin V and propidium iodide (PI). The apoptotic cells were analyzed by flow cytometry. (**D**) Statistical analysis of the apoptotic T cells. The values are represented as the means ± SEM of four independent experiments; * *p* < 0.05 via one-way ANOVA.

Enzyme 3β-HSD converts pregnenolone (P5) to progesterone, which can be further transformed into corticosteroids or androgens. To test the effects of IL4 on the 3β-HSD enzymatic activity, the HT-29 cells were incubated with IL4 for 12 h, and then pregnenolone was added to the medium for 1 h or 2 h. Figure 6A shows that the progesterone levels were higher in the IL4-treated cells than in the control cells, indicating the induction of 3β-HSD activity by IL4. To further examine whether IL4 stimulates the production of GCs, the HT-29 cells were treated with IL4 in the presence of pregnenolone. The conditioned media (CM) were collected and analyzed for the presence of GCs using a GC bioassay as described previously [26]. The HET293T cells were co-transfected with GC receptor (GR)-dependent reporter construct pGRE-tk-Luc and GR expression plasmids, followed by incubation with the cell culture medium from HT-29. Dexamethasone, a synthetic GC, was used as the positive control. As shown in Figure 6B, IL4 treatment significantly increased the GC bioactivity in the presence of pregnenolone. 

GCs are potent inducers of apoptosis, predominantly in T lymphocytes [31]. We then explored whether the CM from the IL4-treated HT-29 cells had the potential to promote T cell apoptosis. The murine splenic cells were isolated and incubated with the HT-29-derived CM, and apoptosis of T cells was monitored by FACS analysis using annexin V and propidium iodide staining. The percentage of apoptotic CD3^+^ T cells cultured in the CM from the IL4-treated cells with P5 was significantly higher than that cultured in the CM from the untreated control (Figure 6C,D). The effect of dexamethasone on the induction of T cell apoptosis was also assessed.

## 3. Discussion

IL4 can induce 3β-HSD activity in various types of human cancer cells [7,8]. In this study, we demonstrated that IL4 greatly increased the *HSD3B1* expression at both the mRNA and protein levels in the HT-29 colon cancer cells. RT-qPCR analysis revealed an over 100-fold increase in the *HSD3B1* mRNA levels as early as 4 h after the IL4 stimulation. As *HSD3B1* is essential for steroid hormone biosynthesis, our results suggest that IL4 may play a crucial role in the regulation of active steroid synthesis in colon cancer cells. 

STAT6 is a key signaling transducer in the IL4 pathway, and two canonical STAT6-binding sequences have been identified in the proximal *HSD3B1* promoter [7]. It was previously shown that STAT6 sites could specifically bind STAT6 activated by IL4 in several cell lines, such as human breast cancer cells and normal human prostate epithelial cells (PrEC) [7,8]. Similarly, our EMSA data demonstrated that STAT6 binds to the STAT6 site after IL4 stimulation in HT-29 cells. In this study, we further verified that STAT6 phosphorylation is rapidly induced by IL4 in HT-29 cells, which is essential for the activation of STAT6 [32]. The inhibition of STAT6 by AS1517499 strongly suppressed the IL4-induced *HSD3B1* expression. Therefore, the activation of STAT6 signaling is important for IL4-regluated *HSD3B1* expression in HT-29 cells.

PI3K participates in the regulation of various biological processes, such as cell survival, growth, metabolism, and immunity [33]. Gingras et al. [34] reported that IL4 induces the phosphorylation of IRS1 and IRS2, leading to downstream PI3K activation, which is crucial for IL4-mediated 3β-HSD activity in ZR-75-1 human breast cancer cell lines. Similarly, we found that the inhibition of PI3K by LY294002 strongly suppressed the mRNA and protein expression of *HSD3B1* by IL4, indicating that the PI3K signaling pathway is also required for IL4-mediated *HSD3B1* expression in HT-29 cells. AKT is the main downstream effector of PI3K. We showed that AKT activation was rapidly induced by IL4, which was blocked by a PI3K inhibitor. Thus, the PI3K–AKT pathway triggered by IL4 plays a pivotal role in *HSD3B1* expression in HT-29 cells. Steroid hormone biosynthesis in steroidogenic cells is mainly controlled by trophic hormones via the cAMP/protein kinase A (PKA) signaling pathway [1]. However, other signaling pathways, such as the protein kinase C, PI3K, and MAPK pathways, have been implicated in the regulation of steroidogenesis [35,36]. Studies have shown that luteinizing hormone (LH) can stimulate the expression of the steroidogenic CYP17A1 enzyme and androgen production in bovine and goat theca cells by activating the PI3K/AKT pathway, although AKT phosphorylation is induced by LH at 12–24 h of LH [37,38]. Melatonin, which exists in the follicular fluid, increases the expression of steroidogenic acute regulatory protein (StAR) and progesterone production in bovine theca cells [39]. Moreover, fibroblast growth factor 9 (FGF9) activates AKT and ERK1/2 to increase progesterone production in mouse Leydig cells [40]. Consistently with these studies, we showed that IL4 activated AKT phosphorylation to promote the *HSD3B1* expression in the HT-29 cells.

Activated AKT phosphorylates several downstream proteins. Glycogen synthase kinase 3 (GSK3) was the first AKT substrate identified [41]. We found that GSK3 positively affected the *HSD3B1* expression because GSK3 inhibitor CHIR99021 and the knockdown of GSK3α or GSK3β significantly suppressed the mRNA and protein levels of *HSD3B1* after 24 h treatment with IL4. However, IL4 causes activation of AKT and results in the inactivation of GSK3 at the early stage, which may explain why the GSK3 inhibitor had no effect on the IL4-stimulated *HSD3B1* mRNA levels at 4 h and 10 h. Therefore, GSK3 may contribute to elevated *HSD3B1* expression during the late stage of IL4 stimulation. GSK3 is a serine/threonine kinase involved in multiple biological processes, such as glucose metabolism, gene expression, development, cell signaling, and cell survival/apoptosis [42,43], but its impact on steroidogenesis is not well-understood. GSK3 plays a key role in the Wnt/β-catenin signaling pathway [44]. A previous study revealed that luteinizing hormone (LH) induces a rise in Ser9 phosphorylation of GSK3β via cAMP/PKA in luteal cells [45]. The inactivation of GSK3β leads to the stabilization and activation of β-catenin, which may influence the expression of steroidogenic acute regulatory protein (StAR) and enhance progesterone secretion in luteal cells [45]. Additionally, Gunosewoyo et al. [46] showed that some GSK3 inhibitors can stimulate progesterone production in MA-10 mouse tumor Leydig cells. Conversely, our results suggest that GSK3 is a novel positive regulator of IL4-induced steroidogenesis and may contribute to steroid production in colon cancer cells.

Our data showed that the PI3K inhibitor treatment resulted in a significant reduction (~91%) in the IL4-stimulated mRNA levels of *HSD3B1* at 4 h, demonstrating that some downstream effectors of PI3K/AKT, rather than GSK3, are important for the acute action of IL4 on *HSD3B1* transcription. mTOR is a major mediator of AKT that controls cell growth, protein synthesis, and energy metabolism [47]. mTORC1 is activated by LH and involved in LH-mediated expression of steroidogenic genes and androgen synthesis in rat theca–interstitial cells [48]. Furthermore, rapamycin, an mTORC1 inhibitor, reduces human chorionic gonadotropin (hCG)/LH-stimulated expression of steroidogenic genes and progesterone production in human granulosa lutein cells [49]. However, rapamycin did not affect the IL4-induced increase in the HSD3B1 protein levels in the HT-29 cells. A similar result was reported in a breast cancer cell line, in which rapamycin had no impact on IL4-induced 3β-HSD activity in ZR-75-1 cells [34]. Thus, further studies are needed to determine which downstream targets of IL4 stimulation in the PI3K/AKT cascade are important for the early expression of *HSD3B1* in HT-29 cells. 

Cancer cells can evade the detection and destruction by the immune system through multiple mechanisms, such as elimination of antigen presentation, suppression of the immune system, and inhibition of T cells [50,51]. Steroid hormones have strong anti-inflammatory and immunosuppressive activities [52,53]. A recent study has revealed that immune T cells from tumors can upregulate the expression of *Cyp11a1*, a key steroidogenic gene, and produce steroids [54]. Inhibition of steroid synthesis in T cells reduces tumor growth in mice, suggesting that T cell-derived steroids may potentially contribute to tumor immune evasion. In particular, cancer cells exhibit a steroidogenic capacity. Colon cancer cells have been found to express steroidogenic genes and produce GCs which can inhibit T cell activation and induce T cell apoptosis [26]. Steroidogenic enzyme 3β-HSD is necessary for the synthesis of all active steroids. In this study, we demonstrated that IL4 induced 3β-HSD activity and promoted the conversion of pregnenolone to progesterone in the HT-29 cells. Additionally, IL4 stimulated the production of bioactive GCs, which potentially enhanced T cell apoptosis. Clinical studies have revealed that increased levels of IL4 are usually observed in various types of tumors, including colon carcinomas [55,56,57]. IL4 may promote tumor growth and dysregulate antitumor immune responses [58,59,60]. IL4 induction of *HSD3B1* expression has been reported in several cancer cell lines [7,8]. Thus, IL4 seems to have a prominent function in the regulation of steroid synthesis, which may contribute to reduced immune suppression in the tumor microenvironment, thereby facilitating tumor growth and metastasis. 

**Figure 7:**
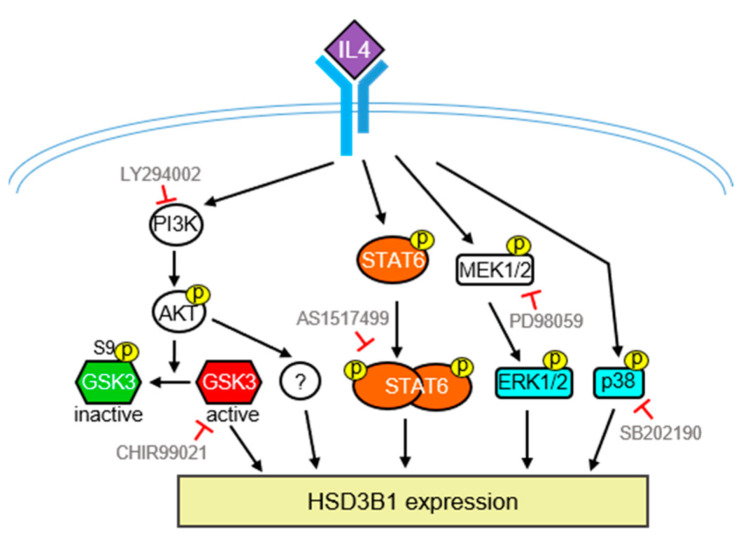
Schematic model of the signaling pathways involved in the IL4-induced *HSD3B1* expression in HT-29 cells. Upregulation of IL4-induced *HSD3B1* expression involves PI3K/AKT, STAT6, ERK1/2, and p38. GSK3 positively regulates IL4-induced *HSD3B1* expression; however, GSK3 is inactivated upon activation of AKT at the early stage of IL4 stimulation. Therefore, GSK3 contributes to an elevated *HSD3B1* expression during the late period of IL4 stimulation. Some downstream effectors of PI3K/AKT important for IL4-induced *HSD3B1* expression have yet to be identified.

In conclusion, as summarized in Figure 7, we demonstrated that IL4 can upregulate *HSD3B1* expression and steroid production in HT-29 human colon cancer cells. Moreover, the STAT6 and PI3/AKT pathways are required for IL4-induced high expression of *HSD3B1* in HT-29 cells. We also found that MAPKs, ERK1/2, and p38 are involved in *HSD3B1* induction by IL4. Therefore, IL4 has the potential to stimulate steroid production, which may be a novel mechanism to achieve IL4-directed antitumor immunity.

## 4. Materials and Methods

### 4.1. Cells and Reagents

The HT-29 and HEK293T cells were grown in Dulbecco’s modified Eagle’s medium (DMEM) containing 10% fetal bovine serum (FBS; Biological Industries, Israel), 100 U/mL penicillin, and 100 μg/mL streptomycin (Thermo Fisher Scientific, Waltham, MA, USA) at 37 °C in 5% CO_2_. Human IL4 was purchased from PeproTech. CHIR99021, dexamethasone, LY294002, pregnenolone, rapamycin, and sodium butyrate were purchased from Sigma-Aldrich. AS1517499 was obtained from MedChem Express. JNKi-V was purchased from Cayman Chemical. PD98059 and SB202190 were purchased from Merck Millipore.

### 4.2. Plasmids

Luciferase reporter construct pGRE-tk-Luc was produced by inserting double-stranded oligonucleotides containing two copies of GC receptor (GR)-binding sequences upstream of the thymidine kinase promoter cloned into pGL3-basic. The expression plasmid for GR (pHA-GR) was provided by Dr. Show-Li Chen (Graduate Institute of Microbiology, National Taiwan University College of Medicine, Taipei, Taiwan).

### 4.3. Western Blotting Analysis

The cells were lysed in a radioimmunoprecipitation assay buffer (50 mM Tris HCl, pH 8, 150 mM NaCl, 1% NP-40, 0.5% sodium deoxycholate, 0.1% sodium dodecyl sulfate, 5 mM EDTA, 1 mM EGTA, 5 mM DTT, 2 mM phenylmethylsulfonyl fluoride, and 10 μg/mL leupeptin) and incubated on ice for 30 min. After centrifugation, the supernatant was collected and subjected to Western blotting analysis. The antibodies used in this study were as follows: HSD3B (sc-30820 or sc-100466; Santa Cruz Biotechnology, TX, USA), STAT6 (Thermo), phospho-STAT6 (Tyr641; Thermo), glyceraldehyde 3-phosphate dehydrogenase (GAPDH; Millipore, MA, USA), β-actin (Sigma-Aldrich, St. Louis, MO, USA ), AKT (Santa Cruz Biotechnology), phospho-AKT (Ser473; Cell Signaling Technology, MA, USA), p-GSK3β (Ser9; Cell Signaling Technology, Danvers, MA, USA), and GSK3β (BD Biosciences, Franklin Lakes, NJ, USA). 

### 4.4. RNA Extraction and Reverse Transcription Quantitative Polymerase Chain Reaction (RT-qPCR)

Total RNA was isolated using the TRIzol reagent (Thermo) and reverse-transcribed into cDNA using a Magic RT Mastermix cDNA synthesis kit (Biogenesis). qPCR was performed using a StepOnePlus machine (Thermo) with a SYBR Green PCR Master Mix (Thermo). The primer sequences used for qPCR are listed in Table 1. Relative quantification of the mRNA levels was performed using the 2^−ΔΔCt^ method and normalized to GAPDH according to the manufacturer’s instructions (Applied Biosystems, Waltham, MA, USA).

### 4.5. Electrophoretic Mobility Shift Assay (EMSA)

The oligonucleotides containing the STAT6-binding site from the human *HSD3B1* promoter were end-labeled with [γ^−32^P] ATP and T4 polynucleotide kinase (NEB), annealed, and purified using Illustra MicroSpin G-25 Columns (GE Healthcare, Chicago, IL, USA). The sense strand sequence of the probe was 5′-TTCCTGTTCCTGGGAAGAATTAGAGATGTA-3′. Nuclear extracts (10 μg) from the HT-29 cells were incubated with the probe in a reaction buffer (20 mM HEPES, pH 7.5, 50 mM KCl, 0.5 mM EDTA, 0.5 mM DTT, 100 μg/mL BSA, 5% glycerol, and 0.5 μg poly (dI-dC)) for 15 min at room temperature. For competition experiments, a 100-fold molar excess of unlabeled oligonucleotides was added to the mixture. For the antibody disruption experiments, 2 μg of anti-STAT6 or the respective isotype antibody was used. DNA–protein complexes were resolved on a 6% non-denaturing polyacrylamide gel (Tris/borate/EDTA), and signals were detected using a Typhoon FLA 9400 PhosphorImager (GE Healthcare Life Sciences).

### 4.6. Short Hairpin RNA (shRNA) Knockdown

shRNA-expressing lentiviral plasmids (pLK O.1-shRNA) were purchased from the National RNAi Core Facility (Academia Sinica, Taipei, Taiwan). GSK3α and GSK3β expression was efficiently silenced by targeting constructs TRCN0000308048 and TRCN000039999, respectively. shRNA construct TRCN0000072223, which is specific for LacZ, was used as the control. To produce lentiviral particles, the pLK O.1-shRNA construct was co-transfected with plasmid pCMV−ΔR8.91 and envelope plasmid pMD.G into the HEK293T cells. The culture media containing lentivirus were harvested and filtered using a 0.22 μm filter at 24 and 48 h post-transfection. The HT-29 cells were incubated with a virus-containing medium with 8 μg/mL polybrene (Sigma-Aldrich). After 48 h of transduction, the cells expressing shRNA were isolated via puromycin selection (5 μg/mL).

### 4.7. Progesterone Measurement

The HT-29 cells were cultured in a serum-free medium with or without IL4 (20 ng/mL). After 12 h, the medium was replaced with a fresh medium containing pregnenolone (1 nM). After 1 or 2 h, the media were collected, and the concentration of progesterone was determined using the enzyme-linked immunosorbent assay (Cayman Chemical Company, Ann Arbor, MI, USA).

### 4.8. Conditioned Medium (CM) Preparation

The HT-29 cells were plated in a 12-well plate or 6 cm Petri dishes. After 24 h, the cells were treated with or without IL4 (20 ng/mL) in fresh DMEM containing 10% charcoal/dextran-treated FBS, and pregnenolone (30 μM) was added as indicated. After 24 h or 48 h, the culture medium was harvested and inactivated at 56 °C for 30 min. After centrifugation, the supernatant was collected and used as the CM.

### 4.9. GC Bioassay

The HEK293T cells were transiently transfected with the GR expression plasmid, pGRE-tk-Luc reporter construct, and control reporter phRLuc using a Turbofect (Thermo Fisher Scientific). After 24 h, the cells were cultured in the CM from the HT-29 cells treated with or without IL4. After 24 h, the cells were lysed, and luciferase activity was analyzed using a Dual-Glo Luciferase Assay System (Promega, Madison, WI, USA). The results were normalized to internal Renilla luciferase activity. 

### 4.10. T Cell Apoptosis Assay

The animal experiments were approved by the National Taiwan University College of Medicine and the College of Public Health Institutional Animal Care and Use Committee. All the methods were performed in accordance with the relevant guidelines and regulations. C57BL/6J mice aged 6–9 weeks were sacrificed using CO_2_. Their spleens were collected and mashed using a strainer (40 μm). The cells were centrifuged at 600× *g* for 5 min and resuspended in 2 mL of a lysis solution (150 mM NH_4_Cl, 10 mM KHCO_3_, and 0.1 mM Na_2_EDTA) for 2 min at 37 °C to remove red blood cells. The cells were added to 30 mL PBS for centrifugation and seeded in a 60 mm dish in the CM of the HT-29 cells as described above. After 16 h of incubation at 37 °C, the cells were collected in a flow buffer (5% FBS and 0.09% sodium azide in PBS) and treated with an Fcγ blocker (BD Biosciences) on ice for 5 min. For T cell identification, the cells were stained with an APC-conjugated anti-mouse CD3e antibody (BD PharMingen) in the dark for 30 min on ice. After washing with the flow buffer, the cells were stained with propidium iodide and annexin V ( Abcam, Cambridge, UK) for 10 min. Apoptotic T cells were analyzed using flow cytometry (BD Biosciences).

### 4.11. Statistical Analysis

Statistical analysis was conducted using Student’s *t*-test or one-way analysis of variance, followed by the Newman–Keuls post-test. The data were analyzed using GraphPad Prism 6 (GraphPad Software, San Diego, CA, USA). Statistical significance was set at *p* < 0.05.

## Figures and Tables

**Table 1:** Primer sequences for RT-qPCR.

Gene	Forward/Reverse Primer
GAPDH	F: 5′-AATCCCATCACCATCTTCCA-3′ R: 5′-TGGACTCCACGACGTACTCA-3′
GSK3A	F: 5′-GCCCACTTCCCCCTCTCTT-3′ R: 5′-GTTGAGAGACGGTTGGATGGA-3′
GSK3B	F: 5′-CGGTGCAGCAGCCTTCA-3′ R: 5′-TGCCGTCCTTGTCTCTGCTA-3′
HSD3B1	F: 5′-CGGCTAACGGGTGGAATCTG-3′ R: 5′-CCCCATAGATATACATGGGTCGTAAG-3′

## Data Availability

Not applicable.

## Supplementary Materials

The supporting information can be downloaded at: https://www.mdpi.com/article/10.3390/ijms232113572/s1.

## Abbreviations

AKT	Protein kinase B
c-AMP	Cyclic adenosine monophosphate
CM	Conditioned medium
EMSA	Electrophoretic mobility shift assay
ERK	Extracellular signal-regulated kinase
GC	Glucocorticoid
GR	Glucocorticoid receptor
GSK3	Glycogen synthase kinase 3
GAPDH	Glyceraldehyde-3-phosphate dehydrogenase
3β-HSD	3β-Hydroxysteroid dehydrogenase/isomerase
HSD3B1	Hydroxy-delta-5-steroid dehydrogenase, 3 beta- and steroid delta-isomerase 1
IL4	Interleukin 4
IRS	Insulin receptor substrate
JNK	c-Jun N-terminal kinase
LH	Luteinizing hormone
mTOR	Mechanistic target of rapamycin
MAPKs	Mitogen-activated protein kinases
MEK	MAP kinase
PI3K	Phosphoinositide-3-kinase
P5	Pregnenolone
shRNA	Short hairpin RNA
PKA	Protein kinase A
StAR	Steroidogenic acute regulatory protein
