# Supplementary material for: HSD3B1 Expression Is Upregulated by Interleukin 4 in HT-29 Colon Cancer Cells via Multiple Signaling Pathways
Source: Int J Mol Sci. 2022 Nov 5;23(21):13572. doi: 10.3390/ijms232113572 (PMC9654614; doi:10.3390/ijms232113572)
Supplement: Supplementary file 1 [file ijms-23-13572-s001.zip › ijms-1992694-supplementary.pdf]

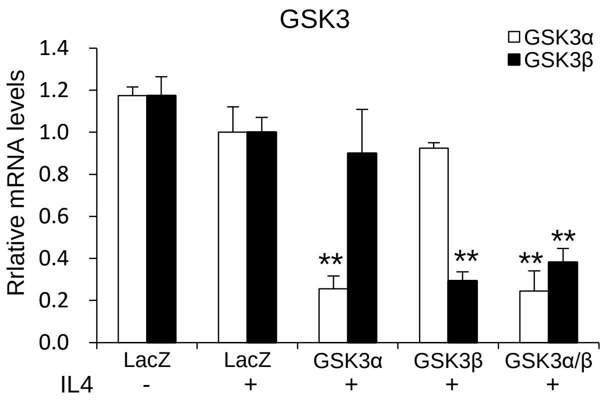

Supplementary Figure S1. Silencing efficiency of shGSK3. HT-29 cells were transduced with shRNAs specific for GSK3α, GSK3β, or LacZ (negative control) for 48 h and exposed to IL4 for 24 h. GSK3α and GSK3β mRNA levels were quantified via RT-qPCR. Values are represented as the mean ± SEM of three independent experiments. $**P < 0.01$ compared with shLacZ+IL4.

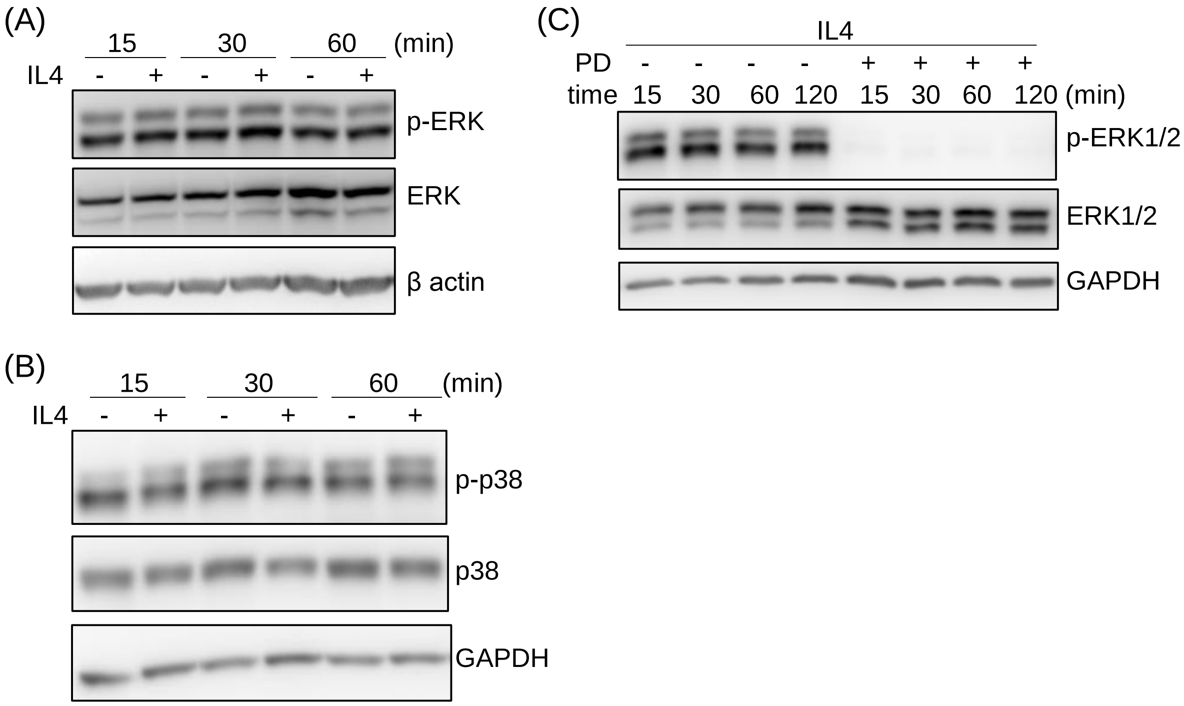

Supplementary Figure S2. Phosphorylation of MAPKs, ERK1/2 and p38, by IL4 in HT-29 cells. HT-29 cells were incubated with IL4 (20 ng/mL) for various time periods. Phosphorylation of ERK1/2 (A) and p38 (B) was analyzed via immunoblotting. (C) Cells were pretreated with 10 μM PD98059 (inhibitor of MEK1/2 upstream of ERK1/2) 30 min before incubation with IL4.
